# The Impact of Ramadan Observance upon Athletic Performance

**DOI:** 10.3390/nu4060491

**Published:** 2012-06-07

**Authors:** Roy J. Shephard

**Affiliations:** 1 Faculty of Kinesiology and Physical Education, University of Toronto, Toronto, ON M5S 1A1, Canada; Email: royjshep@shaw.ca; Tel.: +1-604-898-5527; Fax: +1-604-898-5724; 2 PO Box 521, Brackendale, BC V0N 1H0, Canada

**Keywords:** anaerobic capacity, anaerobic power, aerobic power, fatigue, muscular strength, perceived exertion, vigilance

## Abstract

Ramadan observance requires a total abstention from food and drink from sunrise to sunset for a period of one month. Such intermittent fasting has only minor effects upon the overall nutrition and physiological responses of the general sedentary population. Larger meals are consumed at night and in the early morning. Body mass usually remains unchanged, the total energy intake remains roughly constant, and there is little alteration in the relative consumption of protein, fats and carbohydrates. However, Ramadan observance may be of greater consequence for the training and performance of the competitive athlete, particularly when the festival is celebrated in the hotter part of the year and daylight hours are long, as is the case for the 2012 Summer Olympic Games in London, England. The normal sleeping time then tends to be shortened, and blood sugar and tissue hydration decrease progressively throughout the hours of daylight. Some limitation of anaerobic effort, endurance performance and muscle strength might be anticipated from the decrease in muscle glycogen and body fluid reserves, and a reduced blood glucose may cause a depressed mood state, an increased perception of effort, and poorer team work. This review considers empirical data on the extent of such changes, and their likely effect upon anaerobic, aerobic and muscular performance, suggesting potential nutritional and behavioral tactics for minimizing such effects in the Muslim competitor.

## 1. Introduction

The observance of Ramadan requires a total abstention from both food and drink from sunrise to sunset for a period of one month. Plainly, this form of intermittent fasting is liable to have implications for both training and performance of the competitive athlete. In one survey of 734 16-year-old athletes, 29% concluded that their performance was poorer during Ramadan [1]. Although the consumption of food and drink is unrestricted during the hours of darkness, meals are necessarily reduced to two rather than three or more, with no possibility of taking daytime snacks. One might anticipate both a reduction of total energy intake and a change in composition of the diet, with a late evening meal and/or an early breakfast shortening the normal hours of sleep. Practical problems might be anticipated in terms of a progressive reduction in muscle and hepatic glycogen reserves, a progressive fall of both fluid reserves and blood glucose levels over the hours of daylight, a risk of dehydration over the course of prolonged effort, a reduced availability of metabolites for both anaerobic and endurance activity, and cerebral consequences of a falling blood glucose such as a deterioration of mood state, an increased perception of effort, and a deterioration in cooperation between team members. 

Nevertheless, the magnitude of any adverse effects depends on the type and level of competition, the duration of the daily fast (greater when Ramadan falls in the summer months, and greater when competing at high than at equatorial latitudes) and the temperature and humidity prevailing during the month of Ramadan. The issue is of current relevance, given that the 2012 Olympic Games are taking place in London during the season of Ramadan. Studies examining the physiological effects of Ramadan have commonly been reported in Arabic and Eastern Mediterranean regional journals [2]. Unfortunately, it has not always been clear whether the individuals studied were athletes, and if so the nature of their sport and the level of competition in which they were engaged. In some instances, subjects have shown a substantial decrease in body mass. For practical reasons, most studies have also omitted a control group, thus making it difficult to be certain of influences from seasonal changes in climate and training rather than from the intermittent fasting. 

The effects of Ramadan observance upon body mass index, energy intake, and respective percentages of glucose, fat and carbohydrate in the diet have already had quite extensive discussion [2,3,4,5,6,7,8,9]. In general, there have been only minor changes in these variables. This present brief review focuses upon alterations in various aspects of athletic performance (anaerobic and endurance performance, muscle strength, changes of mood state and perceptions of effort, and a risk of a deterioration in team cooperation). Where possible, the physiological basis for these changes is considered, and tactics are suggested to minimize the adverse impact upon athletes who wish to observe Ramadan while participating in international competition. Information has been sought primarily via the PubMed and HealthStar Ovid data bases. All relevant abstracted reports have been considered from the initiation of these data bases (1946) to February 2012, combining the term “Ramadan” (424 entries) with terms specific to each of the topics under investigation. Additional relevant information has been sought by scanning the reference lists of selected articles and an examination of the author’s own extensive personal files.

## 2. Anaerobic Power and Capacity

A combination of the terms “Ramadan” and “anaerobic” yielded only five papers [10,11,12,13,14], four of which being reports from the same laboratory, with considerable overlap. Several other reports on Wingate test and sprint performance have been added to this list [15,16,17,18,19,20]. 

Souissi and colleagues [12,14] obtained objective Wingate test measurements of peak and mean power output in 12 healthy male physical education students who observed Ramadan during the month of October. Normal patterns of physical activity were maintained over this period, and subjects were prohibited from ingesting any substance that might encourage sleeplessness. The total energy intake and major dietary constituents showed no significant change over Ramadan. All subjects showed “morning” or “intermediate” patterns of arousal; before Ramadan, they slept for 8 h (23:00–7:00), but during Ramadan sleep was typically reduced to 5.5 h (1:30–7:00). The peak power output showed an increase from morning (7:00) to evening (17:00) as anticipated in well-motivated subjects, initially 0.8 W/kg, 0.5 W/kg during the second week of Ramadan, but only a statistically insignificant 0.2 W/kg in the fourth week and 0.4 W/kg 2 weeks after the end of Ramadan. The fatigue index, in contrast, showed increased diurnal variation during Ramadan [12]. The reduction of peak power and increased fatigue in the afternoons could conceivably reflect a cumulative effect of dietary restrictions upon muscle glycogen reserves, but this seems unlikely, given the lack of change in reported food intake. Given the substantial decrease in the time available for sleeping, it seems more reasonable to attribute changes to either a cumulative loss of sleep (with an impact upon circadian rhythms of motivation, arousal and/or perceptions of muscular fatigue) [21,22] or an effect of afternoon hypoglycemia upon motivation. In support of the sleep deprivation hypothesis, Karli and associates [15] found no change of either anaerobic power or anaerobic capacity in Wingate testing of 10 assorted power athletes who maintained their food intake, fluid balance, sleep patterns and training during an October celebration of Ramadan, even though their measurements were made in the afternoon (15:00–17:30).

Chaouachi *et al*. [10] examined the 5, 10 and 30 m sprint performance of 15 young national-level Judo athletes over the course of a Ramadan observance that occurred during the month of November. Normal training patterns were maintained. No restrictions were imposed upon the night-time intake of food during Ramadan; their reported intake of energy and macro-nutrients remained constant, although body fat content decreased by 0.65 kg. With tests performed at 14:00–16:00, no changes of sprint performance were seen over Ramadan.

Kirkendall and associates [17] studied a large sample of 18-year-old junior soccer players (*n* = 85) over an October Ramadan celebration, finding among their sample 32 non-observant players to offer a measure of control to their observations. Overall food intake showed little change during Ramadan, and because the players were resident, training was well-maintained. There was also little change in sleep quality or duration. Water was provided during testing sessions, but “some” of the observant players used this only as a mouthwash. Among the performance tests performed was a succession of seven 30 m sprints, some subjects performing these in the morning and others in the afternoon. Performance in the fastest run was unchanged over either 10 m or 30 m at the second and fourth weeks of Ramadan, but as in the fatigue index studies of Chtouri *et al*. [12], observant subjects showed a slightly greater slowing over the seven sprints (a deterioration of 9.0% at 2 weeks, and 9.3% at 4 weeks, rather than 7.7% before Ramadan), this effect being more marked for morning than for afternoon tests. 

Meckel and associates [18] had rather similar findings in 14–16-year-old top division Israeli soccer players; sprint speed 13 h following breakfast was unchanged relative to pre-Ramadan data, but there was a small statistically significant increase in the effect of fatigue over six 40 m runs during Ramadan (time of year not-specified). Dietary intake, body mass and hours of sleep remained unchanged (although the quality of sleep may have been poorer, since students woke early, ate a pre-dawn breakfast and then attempted to return to sleep), and there was even a small increase in skinfold thicknesses during Ramadan. However, observations were complicated by a change of training; the duration of intense physical activity dropped from 6.4 to 4.5 h/week and physical education classes were cancelled during Ramadan.

One report from Zerguini *et al.* [20] on 55 free-living professional Algerian soccer players noted a significant decrease of sprint-speed during Ramadan, but this group appears to have decreased their habitual physical activity during Ramadan. A second group of 100 junior Algerian soccer players lived in a controlled camp environment [17,19], with 36 non-observant players providing control data. The reported energy intake remained stable, water intake was increased (1.3 L/day), sodium intake and sweating were decreased, there was a small (0.7 kg) decrease of body mass, and average sleep times decreased by 1 h over the 2004 (October) Ramadan observance. In this environment, sprint speed (tested 11 h after breakfast) remained unchanged. Among other reports, Khedder *et al*. [16] noted decreases in 100 m race performance and Faye *et al.* [13] found poorer performance of 200 and 400 m events, associated with a pre-training hypoglycaemia. 

In summary, observations upon anaerobic performance are as yet limited to autumn and winter Ramadan observance in Middle East and Mediterranean regions, with a corresponding shortening of the fast relative to summer months. Under such circumstances, it appears that if sleep patterns are not disrupted and training is maintained, then athletes show little change of anaerobic power or capacity over the month of Ramadan; any tendency to more ready fatigue with event repetition is probably attributable to sleep deprivation or a phase shift in the intake of food, rather than to a cumulative nutritional impairment.

## 3. Aerobic Power

Combination of the terms “Ramadan” and “aerobic” yielded 7 relevant papers, with some apparent overlap of content [8,10,11,18,20,23,24]. Replacement of the term “aerobic” by “physical endurance” or “endurance performance” added a further 4 relevant articles, some from the same research projects [17,25,26,27].

Ramadan and Barac-Nieto [24] measured maximal oxygen intake directly, using a Bruce treadmill protocol. They observed no difference of peak oxygen transport between before Ramadan and after 2 or 4 weeks of fasting, although their subjects were 18 sedentary male government workers rather than athletes. Chaouachi *et al*. [10], also, reported no significant changes of Léger test predictions of maximal oxygen intake during Ramadan in their study of elite Judokas. Likewise, other investigators saw no changes in the performance of submaximal aerobic exercise during Ramadan [8,28]. Sweileh *et al*. [27] noted some decrease of maximal oxygen intake during the first week of Ramadan, associated with dehydration, but values for their subjects, also, had recovered to pre-fast levels by the fourth week of Ramadan. 

Chtouru and colleagues [11,26] used a Yo-Yo intermittent recovery test to examine maximal aerobic power. During Ramadan, the normal improvement in performance during the afternoon was absent; peak power output and peak velocity were decreased and the fatigue index was increased. Kirkendall *et al*. [17] used the Léger shuttle-run; scores on this test deteriorated relative to controls during the second week of Ramadan, but had had returned at least to control values by the fourth week. 

Zerguini *et al*. [20] noted that by the end of Ramadan, there was a 16% decrease in the distance covered by professional soccer players during a 12 min run (as tested at 14:00), with partial recovery 2 weeks later; however, scores on this test, as with other maximal effort tests depend greatly on the individual’s motivation.

Brisswalter *et al.* [23] compared the 5000 m running performance of 9 middle-distance runners who observed Ramadan relative to 9 non-observant controls. Both groups maintained their total energy, water intake, body composition and training during Ramadan, although some of the observant runners increased the proportion of fat in their diet. Observant athletes showed a 5% increase in 5000 m times at the end of Ramadan (hour of testing not specified), but there was no change in their maximal aerobic power or their running efficiency. Brisswalter and colleagues observed some slowing of the oxygen on-transient during Ramadan, and they suggested that this may have contributed to the impairment of performance that they saw. Meckel and associates [18] examined 3000 m run times in 14–16-year-old soccer players, and they also found a small (1%) deterioration relative to times observed before Ramadan. 

Aziz and colleagues [25,29] used a cross-over design, comparing findings during Ramadan with data for the same ten moderately trained men obtained before or after Ramadan. Normal levels of training were maintained throughout. Subjects each ran for 30 min at 65% of maximal oxygen intake, followed by 30 min at maximal speed, with tests performed between 16:00 and 18:00. Nine of the 10 subjects perceived some deterioration in their performance, and the average distance covered was reduced by 4% during Ramadan, although substantial decreases were seen in only 5 of 10 subjects. The authors noted that when slowing occurred, this was usually during the latter phases of the run, possibly pointing to an effect of Ramadan upon glycogen reserves or hydration. In similar vein, Stannard and Thompson [30] found that 4 of 8 men were unable to complete the final 10 min of a 30 min progressive cycle ergometer test during Ramadan. Although most authors consider muscle glycogen reserves sufficient for 90 min of running, at least one study has pointed to an effect upon performance over a shorter time span [31]. In terms of hydration [30], subjects showed a higher urinary specific gravity during Ramadan, but body mass decreased by only a statistically insignificant 0.8%, less than the amount normally thought to influence performance. Hours of sleep were also shortened during Ramadan, and although there was no subjective increase of sleepiness, alertness and concentration were diminished [30]. 

In summary, athletes show no Ramadan-related deterioration in their maximal oxygen intake over a traditional progressive test of perhaps 10 min duration, but with longer bouts of endurance exercise, there is some deterioration of performance as effort continues. It remains unclear whether this is due to poorer motivation, depletion of glycogen reserves, or progressive dehydration.

## 4. Muscle Strength

A literature search based on the terms “Ramadan” and “strength”, “muscle strength”, “muscle force” or “vertical jump” yielded only 2 relevant items [14,32]; 6 additional studies have been identified from personal files [10,17,18,20,23,33].

Bigard *et al*. [32] studied 11 fighter pilots rather than athletes, and their body mass and plasma volume decreased over Ramadan. Under these circumstances, the maximum isometric force of the elbow flexors decreased by 10–12% relative to control data collected 2 months after Ramadan. Muscular endurance at 35% and 70% of maximal force was also reduced by 28 and 22%, respectively. The effects of tissue hydration upon muscle performance are not very consistent [34], but the decrease of muscle function seems in line with what has been observed in wrestlers who fail to rehydrate following the making of a desired weight categorization [35]. Chaouachi *et al*. [36] noted a 4.4% decrease in the plasma volume of Judokas by the end of Ramadan. Brisswalter *et al*. [23] reported a smaller 3.2% decrease of maximal knee extension force in 9 middle-distance runners at the end of Ramadan, when their scores were evaluated relative to those for 9 non-observant controls.

Effects upon muscle performance tests have been even smaller. With tests performed at 17:00, Meckel *et al*. [18] noted a 1.8% decrease of vertical jump in 14–16-year-old soccer players from before to the end of Ramadan; however, these subjects allowed training to decrease during Ramadan. Kirkendall *et al*. [17], Zerguini *et al*. [20] and Chaouachi *et al*. [10] all observed no decrease in vertical jump, squat jump or counter-movement jump scores during Ramadan, although the average power of the judokas during 30 s of repeated jumps did decrease slightly, from 23.4 to 22.4 W/kg. The repeated jumping was final test of the day, when fluid depletion would have been maximal, although the decrement of performance could also reflect a decrease of central drive associated with a fall of blood glucose levels. Observations on 12 female athletes also showed no decrease of vertical jump relative to data collected before and after Ramadan [33].

In summary, if subjects maintain hydration and training, there seems at most a very small decrease in muscle contractile force during Ramadan.

## 5. Cerebral Function

Several reports have examined the impact of Ramadan observance upon the perception of effort [11,12,19,29,37,38], mood-state [10,11,29,39], and vigilance [29,40,41]. As yet, there have been no observations on ability to cooperate with other players in team sports, to carry out highly skilled activities such as skiing or gymnastics, or to engage in detailed planning (as in dinghy sailing).

### 5.1. Ratings of Perceived Exertion

Most observers have used the Borg rating of perceived exertion, and with one exception, Ramadan appears to have had no effect upon perceived effort. Twenty minutes post-exercise, Leiper and colleagues [38] and Zerguini *et al*. [19] compared formal ratings of perceived exertion and subjective impressions of the difficulty of training between 48 soccer players that observed Ramadan and 31 that did not; they found no inter-group differences. Likewise, Aziz *et al*. [29] found no Ramadan-related changes in 10 moderately trained runners. On the other hand, Chtourou and colleagues [11,12] studied the perceptions of groups of 20 and 10 soccer players during both Wingate and aerobic testing; they saw increased ratings of perceived exertion during Ramadan relative to observations made one week before Ramadan.

### 5.2. Mood Profile

Information on mood changes during Ramadan has commonly been obtained from the Profile of Mood States (POMS) questionnaire; where change has been observed, there has been an increase in fatigue scores. Chennaoui and colleagues [39] studied the effects of a November Ramadan observance in 8 middle-distance athletes. Their sample showed a trend towards a decrease of body mass and body fat, with a significant 8% decrease of energy intake by the 21st day of Ramadan. Sleep was also substantially curtailed (by 20% on day 21). The POMS fatigue score was increased by Day 21 of Ramadan, but there were no significant changes in scores for the other 5 POMS scales. Chaouachi *et al*. [10] also reported an increase in the fatigue scores of Judokas, using an 8-item fatigue questionnaire, and Chtourou *et al*. [11] noted an increase of fatigue on the POMS scale in 20 junior male soccer players. However, Aziz *et al*. [29] found no change of mood on the Profile of Mood States in a group of moderately trained subjects.

### 5.3. Vigilance

Roky and collaborators tested various measures of cerebral function before, during and following Ramadan in 10 healthy (but apparently not athletic) subjects [40]. During the month of Ramadan, alertness was decreased at 9:00 and 16:00 but not at 23:00, and reaction time slowed, particularly at the beginning of Ramadan. Tian and associates [41] also examined various aspects of cerebral function in 18 young male athletes. During Ramadan, they found that psychomotor performance and vigilance were actually enhanced at 9:00, but verbal learning and memory were impaired at 16:00, when blood glucose levels were presumably reduced. Aziz *et al*. [29] found no changes of sleepiness during Ramadan, but they also noted that alertness and concentration were decreased during the day time.

## 6. Potential Remedies

Potential tactics to reduce the adverse effects of Ramadan upon athletic performance seem a change in the timing of events, minimization of sleep deprivation, maintenance of blood sugar and hydration, optimization of mood-state, and maintenance of a full training program [42]. Although these various potential measures seem logical suggestions for coaches and trainers, the benefits of few of them have been tested experimentally.

### 6.1. Event-Timing

The simplest counter-measure to avoid the adverse effects of Ramadan observance would seem to modify the timing of events, allowing athletes to compete either early in the morning or late at night. This tactic is commonly adopted during competition between Arabic nations, and it probably counters most problems with the exception of sleep deprivation. However, the times of competition are unlikely to be changed for major international events where most of the contestants are from non-Muslim nations.

### 6.2. Sleep Deprivation

Daytime sleepiness during Ramadan is common, in part because meals tend to be taken late at night and early in the morning, with a disruption of normal sleep patterns [39,43]. One survey of 734 16-year-old athletes found 67% complaining of daytime sleepiness during Ramadan, despite apparently normal patterns of sleep [1]. In contrast, Aziz *et al*. [29] found no increased perceptions of sleepiness during Ramadan.

Any increase of sleepiness is likely to have a negative effect upon sports requiring a strong motor command, concentration or team play [44,45]. If Ramadan falls during the winter months, there seems little reason why an athlete cannot observe Ramadan and still arrange his or her schedule in such a way as to obtain the same period of daily sleep as taken prior to Ramadan. This may be more difficult to arrange when Ramadan is celebrated during the summer months, particularly if competition is scheduled at a latitude where the daylight hours are long. In such a situation, athletes should retire as soon as possible after eating their evening meal, and if it is necessary to eat breakfast before they have obtained 8 h of sleep, they should try to take a nap either after breakfast or at some other time during the day. Provision of a quiet and darkened room will assist sleeping, and it may be useful for the athlete to start adjusting to the proposed sleep regimen before Ramadan begins. 

### 6.3. Maintenance of Blood Sugar

In non-athletic individuals, Ramadan observance has only a minor influence upon resting blood glucose; there is a trend towards a decrease, particularly in the early part of Ramadan, but values remain well within normal clinical limits [46,47]. One report on healthy but not necessarily active adults [48] found a decrease in fasting plasma glucose from 4.9 mmol/L before Ramadan to 3.5 mmol/L; however, this group showed a (possibly deliberate) decrease of food intake during Ramadan. There may be associated decreases in salivary glucose concentrations [49]. If no food is ingested subsequent to an early breakfast, a progressive decrease in blood glucose may be anticipated over the course of the day, and indeed one investigation reported a severe pre-race hypoglycemia [13]. Aziz *et al*. [29] also found a small but statistically significant drop of pre-run blood glucose (from 4.9 to 4.5 mmol/L). Nevertheless, there are considerable inter-individual differences in the rate at which blood glucose falls. No investigators seem to have studied muscle and hepatic glycogen levels during Ramadan. But it seems likely that normo-glycaemia could be prolonged by taking a high carbohydrate diet at night (in order to maximize hepatic and muscle glycogen stores) and eating much of the day’s ration of fat at the Sahur (breakfast) meal (thus delaying gastric emptying). It is also helpful to eat foods with low glycemic index foods at breakfast, in order to prolong the release of sugars into the blood stream [50].

Other possible tactics are to minimize any pre-competitive activity that would otherwise consume blood glucose, and to engage in a spurt of activity immediately before competition (in order to release adrenaline and thus induce glycogenesis from hepatic stores of glycogen). There is also some evidence that the short-term intestinal absorption of carbohydrate can be increased if the gut is “trained” by a high habitual intake of carbohydrate and the diet includes a variety of different carbohydrates [51]. But for those observing Ramadan, a better tactic is probably to encourage the metabolism of fat through endurance training with a high fat diet and a low muscle glycogen content [52,53,54].

### 6.4. Maintenance of Hydration

Problems in maintaining overall body hydration seem commonest at the beginning of Ramadan [27,28,30], and a cumulative loss of body fluid is more commonly seen in sedentary individuals than in athletes [28]. Presumably, experienced competitors learn simple tactics of adaptation. Nevertheless, physically active individuals typically show some increase of serum electrolyte concentrations, even at rest [55], with a statistically significant in urinary specific gravity [29]. Maughan *et al*. noted that the salt intake of observant soccer players was reduced during Ramadan (from 5.4 to 4.3 g/day), whereas a control group showed a small increase of sodium intake [56]. Trabelsi *et al*. [57] also found that the hematocrit values of 12 recreational rugby-seven players immediately after a match were 1.4% higher at the end of Ramadan than before Ramadan. However, the total daily water intake usually shows little change [56], and there may be little change of hemoglobin or hematocrit values [5]. A useful recommendation for an athlete is to drink 600 mL/h of fluid (the normal gastric emptying rate) from the breaking of fast until bedtime, and an additional 1 L at breakfast. 

Serious dehydration is unlikely except in team games performed under hot and humid conditions and during endurance and ultra-endurance events. Shirreffs and Maughan [58] examined young soccer players, finding that the fluid loss over a match was 1.4 L in players observing Ramadan, compared with 1.6 L in those who did not. The sweat loss when running a marathon on a warm day, 3–4 L [59], is of greater concern for homeostasis. The average person stores some 1.5 L of water in association with muscle and liver glycogen, and this water is released into the body as glycogen is metabolized [60]; there is a potential to meet the fluid needs of a soccer match, but a substantial deficit is likely during a marathon run. There is thus a need for study of distance runners during Ramadan. Water reserves could theoretically be as much as doubled if a high carbohydrate diet allowed maximization of pre-game glycogen reserves. Other potential tactics to improve fluid balance include pre-loading of the body with fluid and salt immediately before dawn (some added salt seems desirable, given observations of a reduced intake during Ramadan), minimization of pre-competitive activity, pre-event cooling of the body [61], minimizing pre-event sweating (competition should be awaited in a cool room, with a fan or air conditioner if possible), avoiding radiant heat from the sun, and the wetting of clothing if the environment is very hot. Internal pre-cooling is the most effective method of reducing pre-race core temperatures, but the ingestion of cold water or ice cream is not an option for those who are observing Ramadan. External cooling by immersion in cold water must be used judiciously, because it eliminates the benefits associated with a “warm-up,” with a slowing of metabolic processes, competitive speed and power [62].

### 6.5. Optimization of Mood-State

Athletes commonly report feelings of fatigue and poor performance during Ramadan [39]. Depending on the competitor’s personality, simple psychological tactics are thus required to optimize mood-state and enhance cooperation with other team members. 

### 6.6. Maintenance of Training

Some athletes allow their level of training and/or their habitual activity to decline during Ramadan, with a resulting loss of peak condition. Subjective data for 734 young competitors (average age 16 years) found 20% of this sample expressing the opinion that the quality of their training had decreased during Ramadan. However, this is not a necessary change; training can be maintained [10,15,17,36,63,64,65]. Kordi and associates [66] had 14 athletes train 1 h before Iftar (the evening meal during Ramadan), and 20 other athletes to train 3 h after Iftar; both groups accomplished their normal training routine without any adverse effect upon agility or explosive muscle force. Nevertheless, coaches and trainers should ensure an appropriate periodization of training to avoid over-reaching, particularly if competitors are complaining of increased fatigue, and body mass should be monitored carefully if this seems to be decreasing during Ramadan [10]; it may also be possible to exploit Ramadan to include some desired tapering of training [65]. An appropriate pattern of nutrition is important if cellular adaptations to a given volume of training are to match those seen before Ramadan [67]. A small intake of protein or amino acids immediately prior to strength training facilitates he development of a positive nitrogen balance [68,69], and the muscle glycogen content also affects gene expression in active muscles [70]. Protein synthesis is encouraged by ingestion of 20 g of high quality protein immediately following resistance exercise [71]. Ideally, the protein should be taken immediately following exercise [72,73], although benefit has been reported if food is ingested either before or as late as 3 h following training [74,75]. The practical implication for athletes who are observing Ramadan is that training is best undertaken around the time of their morning or evening meal [29]. 

## 7. Conclusions

Research conducted to date shows relatively minor effects of Ramadan observance upon athletic performance, health or safety, provided that the competitor seeks to maintain his or her intake of energy and fluids, continues a normal pattern of training, and seeks 8 h of sleep per night. Some fatigue seems likely during repeated performance of sprints, times are slower for long distance runs, there is commonly a small decrease of muscle force, and subjects tend to complain of sleepiness and fatigue. Although small, many of these effects are sufficient to influence the outcome of a close competition. Moreover, observations to date have only been completed in years when Ramadan falls during the winter months. Studies are needed when Ramadan occurs during the summer, with higher environmental temperatures and longer hours of daylight. Further, information is still required upon changes of performance during endurance and ultra-endurance events, a possible loss of cooperation in team sports, a deterioration of performance in events requiring prolonged concentration and forward planning, and there are possible dangers from loss of vigilance during the performance of activities such as skiing and gymnastics that require very rapid adjustments of speed and posture.
